# A highly efficient human cell-free translation system

**DOI:** 10.1261/rna.079825.123

**Published:** 2023-12

**Authors:** Nikolay A. Aleksashin, Stacey Tsai-Lan Chang, Jamie H.D. Cate

**Affiliations:** 1 Innovative Genomics Institute, University of California-Berkeley, Berkeley, California 94720, USA; 2 Department of Molecular and Cell Biology, University of California-Berkeley, Berkeley, California 94720, USA; 3 Department of Chemistry, University of California-Berkeley, Berkeley, California 94720, USA

**Keywords:** ribosome, cell-free translation system, protein synthesis

## Abstract

Cell-free protein synthesis (CFPS) systems enable easy in vitro expression of proteins with many scientific, industrial, and therapeutic applications. Here we present an optimized, highly efficient human cell-free translation system that bypasses many limitations of currently used in vitro systems. This CFPS system is based on extracts from human HEK293T cells engineered to endogenously express GADD34 and K3L proteins, which suppress phosphorylation of translation initiation factor eIF2α. Overexpression of GADD34 and K3L proteins in human cells before cell lysate preparation significantly simplifies lysate preparation. We find that expression of the GADD34 and K3L accessory proteins before cell lysis maintains low levels of phosphorylation of eIF2α in the extracts. During in vitro translation reactions, eIF2α phosphorylation increases moderately in a GCN2-dependent fashion that can be inhibited by GCN2 kinase inhibitors. This new CFPS system should be useful for exploring human translation mechanisms in more physiological conditions outside the cell.

## INTRODUCTION

Cell-free protein synthesis (CFPS) systems have become increasingly important to advance biotechnological and fundamental research (Gregorio et al. 2019). In CFPS systems that use cell-free extracts, an mRNA of interest can be translated in conditions that recapitulate protein biosynthesis in vivo and reveal insights into the translation process. Cell extract-based translation systems can also be used to overcome the inherent limitations of cell-based experiments by removing cellular membrane and natural homeostasis mechanisms that prevent the screening of many translational parameters. Whereas cellular growth requires defined conditions, cellular extracts may tolerate substantial genetic or proteomic manipulation and unnatural or toxic components (Shimizu et al. 2001). In addition to functional and structural studies (Spirin et al. 1988), proteins expressed in cell-free extracts can be used for selective and site-specific labeling, stabilization of membrane proteins in a soluble state, and optimizing the production of toxic proteins (Li et al. 2014). In vitro translation systems also allow high-throughput screening of thousands of individual proteins translated from mRNA libraries (Spirin 2004; Goto and Suga 2021).

To enable high levels of protein synthesis, CFPS systems require supplementation with exogenous amino acids, cofactors (proteins, small molecules, and inorganic ions), and energy sources (Gregorio et al. 2019), in addition to the translation templates provided in the form of mRNA, or DNA in the case of transcription–translation coupled systems. Many organisms have been used successfully as the source for extracts to prepare in vitro translation systems. *Escherichia coli* and wheat germ extracts have the highest protein production yields among the cell-free translation systems and are widely used to produce recombinant proteins (Gregorio et al. 2019). However, these systems often do not recapitulate conditions needed to translate mammalian proteins of interest.

To date, mammalian extracts have been limited to human cell lines and rabbit reticulocyte lysates (RRL). While these mammalian systems may be an adequate model for fundamental translation studies, and can provide a native environment for protein folding and post-translational modification, several shortcomings limit their functionality (Gregorio et al. 2019). Preparing RRL extracts requires labor-intensive maintenance, treatment, and sacrifice of animals, while commercially available lysates are expensive (Penzo et al. 2016). Using rabbits as a source also limits the possibilities for genetic manipulations, including the ability to knock out or enrich specific proteins. Furthermore, RRL derives from a highly differentiated and specialized animal tissue, which limits the scope of its translation regulation mechanisms. Finally, RRL endogenously contains very high concentrations of globin mRNAs, which outcompetes the translation of exogenously added templates unless the RRL is pretreated with a nuclease (Penzo et al. 2016). In contrast, the use of human-cultured cells provides more flexibility, by allowing for precise genetic manipulation and the use of many different cell types (Kobayashi et al. 2011; Rakotondrafara and Hentze 2011; Liaud et al. 2019). However, both RRL and human cell line-derived extracts have relatively low protein yields, which limit their functionality. Therefore, highly efficient human-based translation systems are needed to overcome the limitations of presently used mammalian CFPS systems.

**FIGURE 1.**
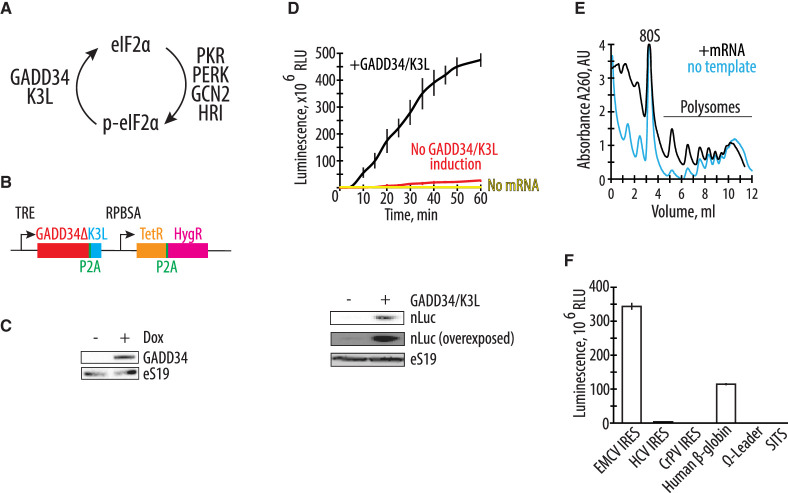
Endogenously expressed GADD34Δ and K3L increase in vitro translation activity of the human cell extract. (*A*) Schematic of the role of GADD34 and K3L in counteracting eIF2α phosphorylation by eIF2α kinases. (*B*) Diagram of the sleeping beauty-based construct used for expression of GADD34Δ (which lacks the amino-terminal 240 amino acids) and K3L, which was integrated into the genome of HEK293T cells. TRE denotes a tetracycline (or doxycycline) responsive promoter that controls the expression of GADD34Δ and K3L, separated by the P2A sequence. The synthetic constitutive promoter *RPBSA* drives the expression of the fusion construct of a tet repressor together with the hygromycin resistance gene, separated by the P2A sequence. (*C*) Western blot showing expression of GADD34Δ in the engineered cell extract upon doxycycline-dependent induction, with ribosomal protein eS19 serving as a loading control. The gel is representative of two independent experiments. (*D*) A time course of nanoluciferase (nLuc) synthesis in the CFPS systems prepared based on the extracts with or without GADD34Δ and K3L expression. All error bars represent one standard deviation of three independent replicates. On the *bottom*, the western blot shows the absolute amount of synthetized nLuc in each of the translation systems with ribosomal protein eS19 serving as a loading control. The gel is representative of two independent experiments. (*E*) Representative polysome profiles of the CFPS based on the extracts with GADD34Δ and K3L expression in the absence or presence of EMCV IRES-containing nLuc mRNA template. (*F*) Nanoluciferase levels from cell-free translation reactions including polyadenylated nLuc mRNAs containing different 5′ UTRs, as indicated. All templates were uncapped, except the human β-globin (*HBB*) 5′ UTR.

A common limitation of presently available mammalian translation extracts is the attenuation of translation initiation due to the phosphorylation of translation initiation factor eIF2 on subunit eIF2α. During translation initiation, eIF2 delivers initiator tRNA to the 40S ribosomal subunit in a GTP-dependent manner. After mRNA start codon recognition, eIF2–GDP is released from the ribosome and is subsequently converted to eIF2–GTP by the guanine nucleotide exchange factor eIF2B. The phosphorylation of eIF2α, which generally occurs in cells during stress (Hinnebusch 2014; Wek 2018; Rios-Fuller et al. 2020), increases the affinity of eIF2 for eIF2B by nearly 100-fold, resulting in the sequestration of eIF2B from the translating pool and inhibition of GDP/GTP exchange (Sonenberg and Hinnebusch 2009). In mammalian cells, four known eIF2α-specific kinases can phosphorylate serine 51 of eIF2α (Fig. 1A). The PKR kinase is activated by double-stranded RNA, as found during viral infection or during in vitro transcription (Ehrenfeld and Hunt 1971; Edery et al. 1989; Anderson et al. 2010; Nallagatla et al. 2011). During stress, eIF2α may also be phosphorylated by PKR-like endoplasmic reticulum kinase (PERK), general control nonderepressible-2 (GCN2) kinase, and heme-regulated HRI kinase (Fig. 1A; Sonenberg and Hinnebusch 2009). Phosphorylation of eIF2α can be bypassed in human cell extracts by the addition of the human GADD34 protein, an eIF2α-specific adapter for PP1 phosphatase, and/or by vaccinia virus K3L protein, a pseudosubstrate for eIF2α-specific kinases that can act as a decoy (Fig. 1A; Davies et al. 1992; Dar and Sicheri 2002; Nonato et al. 2002; Ramelot et al. 2002; Mikami et al. 2006a,b). While eIF2α phosphorylation is thought to be the main limiting factor in CFPS systems, attenuation of the activity of other translation factors has not been studied in depth.

We describe here a highly efficient cell-free translation system based on genetically modified human HEK293T cell extracts. We find that overexpression of a truncated version of GADD34 and K3L in HEK293T cells efficiently reduces the phosphorylation of eIF2α and improves the translation activity of the resulting cellular extract, including robust formation of polysomes on exogenous mRNAs. This system can be used for 5′ m^7^G-capped and internal ribosome entry site (IRES)-containing mRNA templates, in mRNA-dependent or transcription–translation coupled reactions. We also probed the regulation of eIF2α phosphorylation and the phosphorylation status of eukaryotic elongation factor 2 (eEF2) in these extracts, and identified avenues for future optimization of the CFPS system that could enable reconstitution of translation regulatory pathways for biochemical and structural studies.

## RESULTS

### Endogenous expression of GADD34Δ and K3L increases the translational activity of HEK293T cell extracts

To develop a quick and robust method to generate translationally active human extracts, we first tested the hypothesis that endogenously expressed proteins GADD34 and K3L might increase the synthetic activity of the in vitro translation system. We deleted the amino-terminal 240 amino acids of human GADD34, hereafter denoted GADD34Δ, as this deletion allows high levels of GADD34 expression without compromising its enzymatic activity (Mikami et al. 2010b). By using the Sleeping Beauty transposon stable integration system (Kowarz et al. 2015), we engineered the HEK293T human cell line to express both the GADD34Δ and K3L proteins under the control of a doxycycline-inducible promoter (Fig. 1B) (see Materials and Methods). Stable integration of the expression construct minimizes expression variation between different lysates preparations. In addition, a tightly controlled inducible promoter bypasses potential toxicities due to the overexpression of these proteins. After optimization of doxycycline levels, GADD34Δ expression was detected in HEK293T cells without visible toxic effects on cell growth (Fig. 1C).

Efficient in vitro polypeptide synthesis requires substantial energy resources (Spirin et al. 1988; Spirin 2004). To mimic the physiological environment and bypass the potential stringency of nucleotide triphosphates in the CFPS reactions, we supplied the human cellular extract with an energy-recycling system (Shimizu et al. 2001). Although the primary energy consumption of CFPS systems is aminoacyl-adenylate formation (Anderson et al. 2015) and, therefore, the transformation of ATP to AMP and two inorganic phosphate molecules, the creatine kinase commonly used in energy regeneration systems synthesizes ATP from ADP and creatine phosphate (Panthu et al. 2018). To overcome this mismatch, we added rabbit myokinase, which converts the AMP released after tRNA aminoacylation to ADP, the substrate of creatine kinase, by transferring the γ-phosphate from ATP to AMP (Whitford et al. 2007). Furthermore, since several human translation factors use GTP (Schuller and Green 2018), we added nucleotide diphosphate kinase which can maintain the steady-state level of GTP by transferring the γ-phosphate from ATP to GDP (Dumas et al. 1992). Altogether, these three enzymes restore the concentration of ATP and GTP, which is required for efficient cell-free translation.

The engineered HEK293T cell extract with GADD34Δ and K3L was supplemented with an nLuc mRNA, and the in vitro translation was carried out under conditions described in Materials and Methods (Rakotondrafara and Hentze 2011). The synthetic activity of the CFPS system was monitored by the accumulation of enzymatically active nLuc (Hall et al. 2012) and was found to be ∼30-fold more active than extracts from HEK293T cells lacking GADD34Δ and K3L overexpression based on the translation of enzymatically active nLuc (Fig. 1D). Moreover, the new translation system remains synthetically active for much longer periods of time, possibly suggesting the high stability of the mRNA in the CFPS system, and the increased activity of translation factors due to the modified energy regeneration system (Fig. 1D). In the above reactions, magnesium and potassium concentrations were optimized separately in order to maximize the efficiency of in vitro protein synthesis in each extract.

To further characterize the activity of the optimized system, we measured the ability of the engineered cell extract to form polysomes on exogenously added mRNA. Extracts were incubated with polyadenylated nLuc mRNA containing the EMCV IRES, and polysome formation was monitored by sedimentation analysis on 10%–50% sucrose gradients. The polysomes present in the reactions without adding the nLuc mRNA (Fig. 1E, blue line) likely come from the residual translation of the endogenous cellular templates in the extract. Minimizing the background translation by micrococcal nuclease treatment (7) significantly decreases the synthetic capacity of the CFPS (Supplemental Fig. S1). Therefore, for the experiments described here, the cellular extracts used for the preparation of the CFPS were not treated with micrococcal nuclease.

Next, we tested if the CFPS system based on the engineered cell extract can improve the translation of mRNAs with different 5′ UTRs. While the EMCV IRES-containing mRNA helps drive strong protein expression without a 5′ m^7^G-cap structure (Fig. 1F), its large size and complicated RNA secondary structure may limit its applications. We tested the CFPS system with nLuc mRNAs using 5′ UTRs containing the cap-independent HCV IRES (Sun et al. 2013; Jaafar et al. 2016), CrPV IRES (Pestova et al. 2004; Fernández et al. 2014), omega leader (Shaloiko et al. 2004), or synthetic SIST sequence (Mureev et al. 2009). However, we could not identify optimized temperature and ionic conditions for any of these 5′ UTRs that improved their translation to comparable levels as the EMCV IRES (Fig. 1F). The widely used capped *HBB* 5′ UTR (Leppek et al. 2022) shows substantial translational activity in the optimized CFPS system (Fig. 1F), but not as high as with the EMCV IRES.

### The activity of engineered HEK293T cell extracts is comparable to a HeLa-based in vitro translation system

**FIGURE 2.**
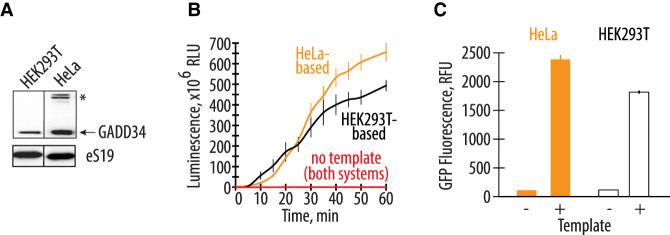
Endogenously expressed GADD34Δ and K3L increase translational activity comparable to the addition of exogenously expressed accessory proteins. (*A*) Western blot showing the amount of the GADD34Δ expressed in the engineered HEK293T cells and supplemented in the HeLa-based commercial translation system. The asterisk indicates a nonspecific band in the HeLa extract. The gel is representative of two independent experiments. (*B*) A time course of nLuc synthesis in the CFPS systems prepared based on the engineered HEK293T cell extract and HeLa-based extract with recombinant GADD34Δ and K3L supplement. All error bars represent one standard deviation of three independent replicates. (*C*) Cell-free synthesis of GFP in the two translation systems. Orange bars represent the HeLa-based extract with exogenous GADD34Δ and K3L added, while the white bars represent the engineered HEK293T cell extract with endogenously expressed GADD34Δ and K3L proteins. All error bars represent one standard deviation of three independent replicates.

The expression and purification of recombinant GADD34Δ and K3L proteins add labor and cost to preparing CFPS systems (Mikami et al. 2006b, 2010b). Therefore, engineering human cell lines to express these recombinant proteins should reduce the cost and time required to prepare extracts for CFPS. Consequently, we compared the translational activity of a commercially available CFPS system with cell-free translation systems prepared in-house. The commercially available system based on the S3 HeLa cell extract contains ectopically purified GADD34Δ and K3L accessory proteins (Fig. 2A). The homemade reaction was based on the HEK293T-based extract expressing GADD34Δ and K3L and contained all the required supplements (see Materials and Methods). The two translation systems were set up in parallel and in the same volume to test their activity, using nLuc or GFP activity assays to monitor the translation of EMCV IRES-containing and polyadenylated mRNAs encoding these proteins. We used nLuc mRNA prepared and added exogenously to the translation reactions, or in separate reactions monitored GFP expression driven from a plasmid in a coupled transcription–translation reaction (see Materials and Methods). When compared to engineered cell extracts prepared as described above, the translational activity of the commercially available HeLa translation system is not substantially higher than the in-house prepared CFPS system prepared from the engineered HEK293T cells (Fig. 2B,C). Thus, the overexpression of the GADD34Δ and K3L accessory proteins in human cell lines rather than subsequent addition to cell extracts effectively promotes CFPS activity to the same extent while significantly reducing preparation time and cost (Mikami et al. 2006b, 2010b).

### Cellular overexpression of the GADD34Δ and K3L proteins protects eIF2α from phosphorylation

**FIGURE 3.**
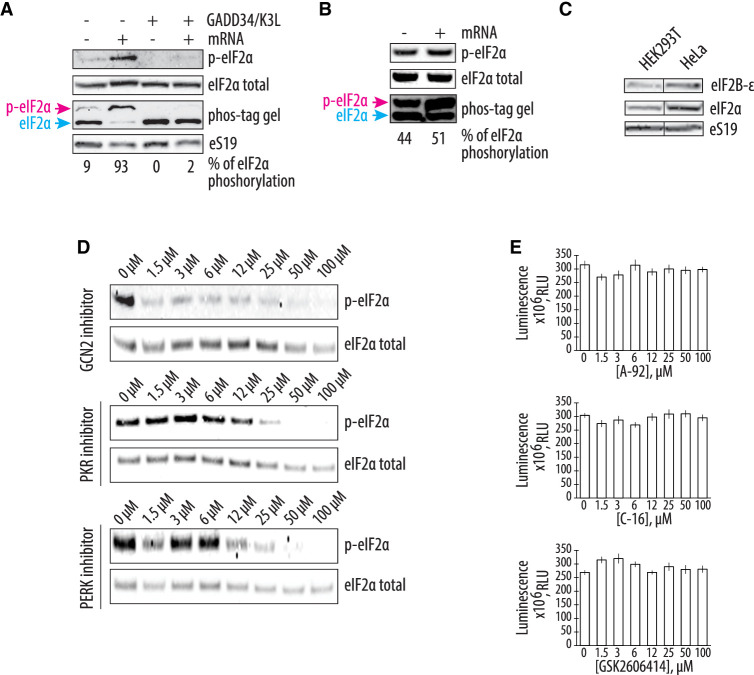
GCN2 kinase is responsible for the residual phosphorylation of eIF2α during CFPS in the engineered HEK293T extract. (*A*) Western blots for phosphorylation of eIF2α in the HEK293T-based CFPS systems with and without GADD34Δ and K3L expression. The uncapped EMCV IRES-containing polyadenylated mRNA was used to drive the synthesis of nLuc. (*B*) Induction of eIF2α phosphorylation in the HeLa-based commercial translation system supplemented with the same mRNA. For both *A* and *B*, the blue arrow indicates the nonphosphorylated form of the eIF2α, while the magenta arrow indicates the phosphorylated form on the Phos-tag gels. The western blots *above* the Phos-tag gels used normal SDS–PAGE gels sequentially blotted with anti-eIF2α and anti-P-Ser51 eIF2α antibodies. For both *A* and *B*, the gels are representative of two independent experiments. The percentage of eIF2α phosphorylation, based on the Phos-tag gels, is indicated *under* the gels (see Materials and Methods). (*C*) Western blot showing the amount of the eIF2B-ε and eIF2α in the HEK293T and HeLa cellular extracts. The gel is representative of two independent experiments. (*D*) The GCN2 but not PKR or PERK kinase inhibitor protects eIF2α from phosphorylation during CFPS. The compound A-92 (Brazeau and Rosse 2014) was used as a GCN2 kinase inhibitor, C-16 (Jammi et al. 2003) for PKR kinase inhibition, and GSK2606414 (Axten et al. 2012) as an inhibitor of PERK kinase. The concentrations of the eIF2α-specific kinase inhibitors are indicated. Gels are representative of two independent experiments. (*E*) Cell-free synthesis of nLuc in different concentrations of the eIF2α-specific kinase inhibitors. All error bars represent one standard deviation of three independent replicates.

The increased translational activity of the CFPS system based on the engineered cell extract seen above is consistent with the role of GADD34Δ and K3L in preventing phosphorylation of eIF2α in the in vitro translation reactions. To test this hypothesis, we probed the phosphorylation state of eIF2α in CFPS systems based on cell lysates from the engineered HEK293T cells with and without GADD34Δ and K3L expression. We first used Phos-tag gels (Kinoshita et al. 2006; Kinoshita and Kinoshita-Kikuta 2011), which separate the phosphorylated and nonphosphorylated forms of eIF2α during gel electrophoresis. Western blots of eIF2α revealed the baseline level of phosphorylated eIF2α in the CFPS translation systems with and without GADD34Δ and K3L expression was similarly low after 1 h of incubation, even though endogenous mRNAs were not removed (Fig. 3A). However, addition of exogenous mRNA to CFPS reactions significantly increased the amount of the phosphorylated eIF2α in extract without GADD34Δ and K3L expression (up to 93% of eIF2α) compared to the small increase seen in engineered cell extracts containing GADD34Δ and K3L (∼2%) (Fig. 3A). This increase was independent of whether the 5′ UTR of the mRNA was cap-dependent (*HBB* 5′ UTR) or used the long and highly structured EMCV IRES (Supplemental Fig. S2). Remarkably, the level of phosphorylated eIF2α was high in the translationally efficient system based on the HeLa extract supplemented with recombinant GADD34Δ and K3L proteins (∼44% and ∼51% phosphorylation of eIF2α without and with exogenous mRNA added, respectively) (Fig. 3B; Supplemental Fig. S2). We also confirmed that the HEK293T and HeLa cellular extracts have the same relative levels of eIF2α and eIF2B (Fig. 3C), consistent with published quantitative mass proteomics studies (Liu et al. 2021), suggesting that eIF2B sequestration of eIF2 may not function correctly in the HeLa cell extract. These results clearly show that overexpression of the GADD34Δ and K3L proteins in HEK293T cells prevents eIF2α phosphorylation more efficiently compared to the ectopically added recombinant proteins (Fig. 3A,B); even though these proteins are added at roughly the same concentration to the two CFPS systems (Fig. 2A).

Next, we asked whether the residual eIF2α phosphorylation in the CFPS system based on the engineered HEK293T cell extract was mediated by a particular eIF2α-specific kinase and whether inhibition of this kinase could affect the protein synthesis activity of the system. Although the four known eIF2α-specific kinases are each activated by specific stress response signals, these signaling pathways might be dysregulated in the context of cellular extracts. For example, GCN2 kinase might be activated by the supplementation of the system with uncharged bovine total tRNA (see Materials and Methods). To test this idea, we checked the phosphorylation state of eIF2α in CFPS reactions pretreated with different eIF2α-specific kinase inhibitors (Fig. 3D). We used GCN2 kinase inhibitor A-92 (IC_50_ = 300 nM; Brazeau and Rosse 2014), PKR-specific inhibitor C-16 (IC_50_ = 210 nM; Jammi et al. 2003), and PERK kinase inhibitor GSK26006414 (IC_50_ = 0.4 nM; Axten et al. 2012). Only the GCN2-specific kinase inhibitor prevented eIF2α phosphorylation at a concentration near its IC_50_ value, whereas the PERK and PKR-specific kinase inhibitors failed to prevent eIF2α phosphorylation, even at concentrations far above their IC_50_ values (Fig. 3D). While this result revealed the likely cause of eIF2α phosphorylation in the cell-free protein expression reactions, the decrease in eIF2α phosphorylation did not increase the translational activity of the engineered cell extract-based human translation system (Fig. 3E), indicating that eIF2α phosphorylation is not the only limiting factor in the new CFPS system.

### Additional factors may slow down translation during the elongation stage of the CFPS

**FIGURE 4.**
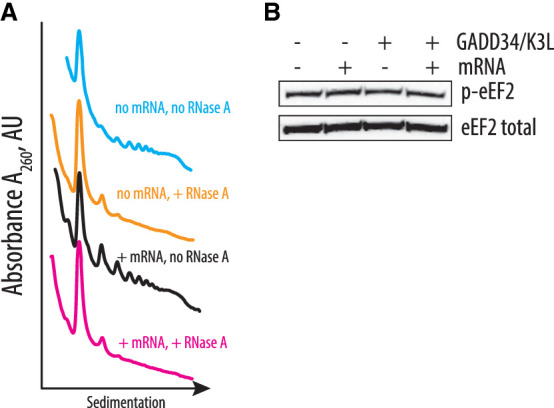
Identification of possible additional CFPS-limiting factors. (*A*) Sucrose gradient sedimentation analysis reveals the presence of disomes and trisomes in the CFPS system based on the extract from the engineered HEK293T cell line, independent of exogenously added mRNA. (*B*) Phosphorylation of eEF2 on T56 in the different translation systems. The capped *HBB* and uncapped EMCV IRES-containing polyadenylated mRNAs were used to drive the synthesis of nLuc. The gels are representative of two independent experiments.

Although upon GADD34Δ and K3L overexpression, eIF2α phosphorylation levels do not exceed a few percent (Fig. 3A), the absence of a correlation between eIF2α phosphorylation and translational activity made us consider what other factors may limit the translation activity of the CFPS system. One potential limitation of the CFPS synthetic activity is the activation of ribosome quality control (RQC) mechanisms due to defective translation in the cellular extract and collisions of translating ribosomes resulting in the formation of the disome and trisome particles (Schuller and Green 2018; Sinha et al. 2020). To probe the formation of such particles in the CFPS extracts, extracts were incubated with or without polyadenylated nLuc mRNA containing the EMCV IRES, digested with RNase A, and the formation of the nuclease-resistant disomes was monitored by sedimentation analysis on 10%–50% sucrose gradients (Fig. 4A). Consistent with our hypothesis, translation of the residual cellular templates results in the formation of nuclease-resistant disomes and trisomes (Fig. 4A, orange line), which may activate ribosome-dependent quality control mechanisms. Notably, translation of the nLuc template does not further increase disome and trisome formation, despite the substantial increase of the polysomes (Fig. 4A, magenta and black lines, respectively). We speculate that the appearance of the disomes and trisomes due to the residual and possibly aberrant translation of the endogenous templates might be the reason for the activation of the GCN2 kinase (Fig. 3), as recently described (Wu et al. 2020).

Cellular translation might be regulated not only during initiation and by RQC, but also during elongation. For example, it is possible that any degree of inhibitory phosphorylation of translation factors could potentially decrease the activity of in vitro translation systems. We hypothesized that CFPS might also be repressed by the phosphorylation of threonine 56 of eEF2, which prevents eEF2 binding to the ribosome and globally down-regulates translation in human cells during stress conditions (Ovchinnikov et al. 1990; Price et al. 1991; Hizli et al. 2013). Elongation factor eEF2 translocates the tRNAs and mRNA through the ribosome and, therefore, is essential during each round of translation elongation. Western blot analysis with antibodies specific to the T56 phosphorylated form of eEF2 revealed phosphorylation of eEF2 upon in vitro translation in all tested CFPS extracts, independent of the presence of the GADD34Δ and K3L proteins (Fig. 4B). Although phosphorylation does not increase upon adding exogenous nLuc mRNA, it may still decrease the synthetic capacity of the CFPS reactions by reducing the concentration of active eEF2 and, therefore, extending the time of each elongation cycle.

## DISCUSSION

Here, we describe the preparation of a highly active CFPS system derived from human cells engineered to express GADD34Δ and K3L, which minimize eIF2α phosphorylation in the extract. We also implement a new energy regeneration system informed by the nucleotide pools that result from the different steps in translation. One of the most significant advantages of the translational system described here is that all components of the translational machinery are derived from human sources, apart from the added bovine tRNA. While previous results in Chinese hamster ovary (CHO) cells that transiently overexpressed GADD34Δ also showed increases in the translational activity in a CFPS system (Heide et al. 2020), the use of CHO cells may introduce confounding variables to the study of aspects of human translation that are not conserved. Stable integration of the recombinant genes for GADD34Δ and K3L in the genome as described here should also enable more controlled preparation of cell extracts without complications due to transient transfection on cell viability (Heide et al. 2020). One of the possibilities for the future improvement of the CFPS system would be the usage of the H47R mutant form of K3L, which was shown to possess higher inhibitory activity against PKR kinase compared to WT K3L (Kawagishi-Kobayashi et al. 1997; Elde et al. 2012). In the case of human-derived CFPS systems, we find that HeLa translation systems can achieve high levels of translation (Fig. 2). Although this in vitro translation system is supplemented with individually purified GADD34 and K3L, the high levels of eIF2α phosphorylation seen in the HeLa extracts (Fig. 3B) indicate that HeLa translation extracts are highly dysregulated and are not suited to the study of mechanisms of translation regulation. Moreover, the composition of the commercially available translation mix is not fully defined, impeding its facile manipulation required for foundational investigations of human translation. Finally, purification of GADD34Δ and K3L from bacteria has proven to be challenging (Mikami et al. 2010b). Thus, the changes to lysate preparation and the translation reactions outlined here should enable the study of human translation under more physiological conditions.

The overexpression of GADD34Δ and K3L prior to cell lysis helps alleviate the inhibitory effect of eIF2α phosphorylation and supports high levels of translation exemplified by the formation of robust polysomes (Fig. 1E). Furthermore, cellular expression of these two proteins increases the reproducibility of the in vitro translation experiments by decreasing the initial phosphorylation of eIF2α upon lysis (Fig. 3A). Although our translational system is not treated with micrococcal nuclease to deplete endogenous mRNAs, the extract preserves the integrity of all translation machinery components resulting in high levels of translation (Fig. 2; Supplemental Fig. S1), and can be used with both m^7^G-cap dependent (*HBB* 5′ UTR) and IRES-dependent mRNAs (EMCV IRES) (Fig. 1F). The physiological phosphorylation status of eIF2α may allow the study of the effect of different factors known to influence mRNA translation, including the roles of translation initiation factors and the effects of RNA sequence and secondary structure on translation initiation (Levy et al. 1991; Hentze and Kühn 1996; Asano et al. 2000; Hsieh et al. 2012; Sokabe et al. 2012; Lee et al. 2015; Xue and Barna 2015; Xue et al. 2015; Pulos-Holmes et al. 2019). The fact that the present CFPS system produces polysomes in a robust manner may also allow in vitro studies of the mRNA circularization model of translation (Wells et al. 1998; Pelletier and Sonenberg 2019). Finally, maintaining the physiological state of the eIF2α should also make the human-derived CFPS system valuable for assessing translational events requiring a high level of CFPS translational activity such as translational fidelity during alternative decoding events (Leppek et al. 2022), programmed ribosomal frameshifting (Atkins et al. 2016), and RQC pathways (Dever et al. 2018; Schuller and Green 2018).

The CFPS system described here provides substantial improvements to the use of in vitro cellular lysates for the study of human translation. However, future improvements are likely possible within this system. For example, it could be useful to find ways to deplete endogenous mRNAs from the extract, that is, to eliminate the formation of residual disomes and trisomes (Fig. 4A) without the use of nucleases. Additionally, we observe phosphorylation of elongation factor eEF2 in all the extracts we examined (Fig. 4B). While additional biochemical analysis is required to quantify the percent phosphorylation of eEF2, the extracts might be further improved by decreasing eEF2 phosphorylation at residue T56 by using eEF2 kinase inhibitors (Kodama et al. 2021). The approaches described here could also be used to prepare in vitro cell-free translation systems from other cell types, including immortalized cells derived from different tissues. It may also be possible to prepare CFPS systems from primary cells derived from healthy individuals as well as from patients suffering from diseases associated with translation defects, including rare ribosomopathies (Mills and Green 2017; Kampen et al. 2020). Efforts to reduce the number of cells needed to prepare the engineered CFPS system described here would be required but could provide unprecedented opportunities to study the molecular mechanisms underlying human translation regulation.

## MATERIALS AND METHODS

### Cloning of the pSBtet-GADD34Δ/K3L expression plasmid

The plasmid backbones were prepared by SfiI (NEB) restriction of the pSBtet-Hygro vector (Addgene, 60508). The DNA fragments needed to assemble the constructs encoding GADD34Δ and K3L were PCR amplified using Q5 High-Fidelity DNA polymerase (NEB) from plasmid templates (gift of A. Pulk). For the amplification of the GADD34Δ fragment, the following primers were used:

Fwd-GADD34Δ, 5′-TACCACTTCCTACCCTCGAAAGGCCTCTGAGGCCACCATGAAAGGAGCCAGGAAGACCTCCGTGT-3′
Rev-GADD34Δ, 5′-GCCAGGATTCTCCTCGACGTCACCAGCCTGCTTCAGCAGGCTGAAGTTAGTAGCGCCACGCCTCCCACTGAGGCTCAGG-3′.
For the amplification of the K3L fragment, the following primers were used:
Fwd-K3L, 5′-TGGTGACGTCGAGGAGAATCCTGGCCCCTTAGCCTTTTGTTATTCGCTCCCTA-3′
Rev-K3L, 5′-CATGTCTATCGATGGAAGCTTGGCCTGACAGGCCTCACTGGTGGCGACACATACGTTTATAA-3′.
The pSBtet-GADD34Δ/K3L expression plasmid was constructed by Gibson assembly (Gibson et al. 2009) using the two PCR fragments and the vector backbone. The final sequence was verified by the full plasmid sequencing and the plasmid made available through Addgene (#196136).

### Cell lines and culture conditions

HEK293T cells with the pSBtet-GADD34Δ/K3L construct inserted in the genome were grown in DMEM media (Gibco) supplemented with 10% Tet-system approved FBS (Gibco), penicillin (100 µg/mL) (Gibco), streptomycin (100 µg/mL) (Gibco), and 2 mM GlutaMax (Gibco). Cells were grown at 37°C in 5% carbon dioxide and 100% humidity. Induction of GADD34Δ and K3L expression prior to cell extract preparation is described below.

### Generation of the HEK293T cell line with a stably integrated GADD34 Δ1-240/K3L construct

A stable cell line for endogenous expression of GADD34 Δ1–240 (GADD34Δ) and K3L was generated following the procedure described previously (Kowarz et al. 2015). Using Amaxa SF Cell Line 4D-Nucleofector X Kit (Lonza), wild-type HEK293T cells were transfected with a transposase-encoding plasmid (pSB100X) and the pSBtet-GADD34Δ/K3L construct (see above). After 2 d of recovery, nucleofected cells were selected with 300 µg/mL hygromycin (Thermo Fisher Scientific). A total of three rounds of selection were performed, each with approximately three doublings. Visual observation of the cell culture monitored the efficiency of the selection. After completion of the selection, cells were regularly maintained with 150 µg/mL hygromycin.

### In vitro transcription reactions

In vitro transcription reactions were performed using PCR products generated with primers encoding a flanking T7 RNA polymerase promoter and a poly(A) tail. Reactions were set up, as previously described (Li et al. 2019), with 20 mM Tris-HCl, pH 7.5, 35 mM MgCl_2_, 2 mM spermidine, 10 mM DTT, 1 u/mL pyrophosphatase (Sigma), 7.5 mM of each NTP, 0.2 u/mL RiboLock RNase Inhibitor (Thermo Fisher Scientific), 0.1 mg/mL T7 RNA polymerase, and 40 ng/µL PCR-generated DNA. After 3 h incubation at 37°C, 0.1 u/µL DNase I (Promega) was added to the reactions, which were incubated at 37°C for 30 min to remove the template DNA. RNA was precipitated for 2–3 h at −20°C after adding 0.5× volume of 7.5 M LiCl/50 mM EDTA, and the resulting pellet was washed with cold 70% ethanol and dissolved with RNase-free water. The mRNA was further purified by using a Zymo RNA Clean and Concentrator Kit (Zymo Research) before use in in vitro translation reactions.

DNA templates were amplified from a plasmid containing the corresponding 5′ UTR and the NanoLuc Luciferase coding sequence. Primers used for this amplification added a 30T sequence at the 3′ end to form a poly(A) tail after transcription. The *HBB* 5′ UTR containing mRNA was then capped using Vaccinia Capping Enzyme (New England Biolabs) and 2′-*O*-methylated using Vaccinia 2′-*O* Methyltransferase (New England Biolabs). The IRES-containing mRNAs were uncapped and polyadenylated.

### Preparation of cell extracts for CFPS reactions

The HEK293T-based CFPS system was prepared according to the following procedure. First, approximately 1.2–1.5 million HEK293T cells were seeded per 150 mm plate in HEK293T cell-specific media, described above. The next day, the expression of GADD34Δ and K3L was induced by adding 1 µg/mL of doxycycline (Takara). After one additional day, cells were collected by scraping, washed with ice-cold DPBS (Gibco), and suspended with an equal volume of lysis buffer (10 mM HEPES, pH 7.4, 10 mM KOAc, 0.5 mM Mg(OAc)_2_, and 5 mM DTT). After incubation on ice for 45 min, cells were lysed by pushing through a 1 mL syringe with a 26G needle about 15 times, followed by centrifugation at 15,000*g* for 1 min. After centrifugation, the supernatant was aliquoted to avoid freeze–thaw cycles and flash-frozen in liquid nitrogen.

### In vitro translation reactions

The HeLa extract cell-free translation system was obtained from Thermo Fisher Scientific (catalog #88882) and used according to the manufacturer's instructions. In the case of HEK293T reactions, the optimal concentration of the magnesium and potassium ions was determined for each new preparation of cellular extract, with the conditions below representative of typical final conditions.

Translation reactions with the HEK293T-based CFPS system were set up according to a previously published procedure (Mikami et al. 2010a) with modifications. For a 10 µL mRNA-dependent reaction, 5 µL of cell extract was used in a buffer containing final concentrations of 52 mM HEPES, pH 7.4 (Takara), 35 mM KGlu (Sigma), 1.75 mM Mg(OAc)_2_ (Invitrogen), 0.55 mM spermidine (Sigma), 1.5% Glycerol (Thermo Fisher Scientific), 0.7 mM putrescine (Sigma), 5 mM DTT (Thermo Fisher Scientific), 1.25 mM ATP (Thermo Fisher Scientific), 0.12 mM GTP (Thermo Fisher Scientific), 100 µM l-Arg; 67 µM each of l-Gln, l-Ile, l-Leu, l-Lys, l-Thr, l-Val; 33 µM each of l-Ala, l-Asp, l-Asn, l-Glu, Gly, l-His, l-Phe, l-Pro, l-Ser, l-Tyr; 17 µM each of l-Cys, l-Met; 8 µM l-Trp, 20 mM creatine phosphate (Roche), 60 µg/mL creatine kinase (Roche), 4.65 µg/mL myokinase (Sigma), 0.48 µg/mL nucleoside-diphosphate kinase (Sigma), 0.3 u/mL inorganic pyrophosphatase (Thermo Fisher Scientific), 100 µg/mL total calf tRNA (Sigma), 0.8 u/µL RiboLock RNase Inhibitor (Thermo Fisher Scientific), and 1000 ng mRNA.

For the 10 µL coupled transcription–translation reactions, 5 µL of cell extract was used in a buffer containing final concentrations of 52 mM HEPES, pH 7.4 (Takara), 35 mM KGlu (Sigma), 4 mM Mg(OAc)_2_ (Invitrogen), 0.55 mM spermidine (Sigma), 1% glycerol (Thermo Fisher Scientific), 0.7 mM putrescine (Sigma), 5 mM DTT (Thermo Fisher Scientific), 1.25 mM ATP (Thermo Fisher Scientific), 0.83 mM each of UTP, CTP, and GTP (Thermo Fisher Scientific), 100 µM l-Arg; 67 µM each of l-Gln, l-Ile, l-Leu, l-Lys, l-Thr, l-Val; 33 µM each of l-Ala, l-Asp, l-Asn, l-Glu, Gly, l-His, l-Phe, l-Pro, l-Ser, l-Tyr; 17 µM each of l-Cys, l-Met; 8 µM l-Trp, 20 mM creatine phosphate (Roche), 60 µg/mL creatine kinase (Roche), 4.65 µg/mL myokinase (Sigma), 0.48 µg/mL nucleoside-diphosphate kinase (Sigma), 0.3 u/mL inorganic pyrophosphatase (Thermo Fisher Scientific), 100 µg/mL total calf tRNA (Sigma), 0.8 u/µL RiboLock RNase Inhibitor (Thermo Fisher Scientific), 11 µg/mL T7 polymerase, and 100 ng mRNA.

The HEK293T-based translation reactions were incubated for 60 min at 32°C, and nLuc activity was monitored using the Nano-Glo Luciferase Assay Kit (Promega) in a Microplate Luminometer (Veritas). To account for batch-to-batch variability, all assays presented in a given figure were carried out using the same in vitro translation extract and the same preparation of mRNAs.

For the in vitro translation of GFP experiments, coupled transcription–translation reactions based on the HeLa, and HEK293T extracts were supplemented with 50 ng/µL pCFE1-GFP plasmid (Thermo Fisher Scientific), according to the manufacturer's protocol. After 3 h of incubation, reactions were transferred directly into a black 384-well plate with clear bottom (Greiner). The GFP fluorescent signal was measured using a Tecan Spark plate reader at ex/em: 482/512 nm.

For the kinase inhibition experiments, cell-free translation systems were pretreated with the indicated concentrations of A-92 (Axon Medchem), C-16 (Cayman Chemical), or GSK2606414 (Axon Medchem) inhibitors prior to initiating the translation reactions.

### Micrococcal nuclease treatment of the HEK293T human cell lysates

Micrococcal nuclease treatment of human cell lysates was carried out according to Rakotondrafara and Hentze (2011). Briefly, before setting up the CFPS reaction, cell extract was supplemented with micrococcal nuclease (NEB) at final concentrations of 10, 15, or 20 u/mL and 0.75 mM of calcium chloride (Sigma). After incubation at 25°C for 7 min, the nuclease reaction was stopped by the addition of EGTA (Sigma) solution at a final concentration of 3 mM.

### Polysome profiling

Lysates containing ∼100 pmol of ribosomes (1 A_260_ = 20 pmol) were resolved through 10%–50% sucrose gradients (20 mM HEPES-KOH, pH 7.4, 100 mM KCl, 5 mM MgCl_2_, 100 µg/mL cycloheximide [Sigma], 0.5 mM DTT [Thermo Fisher Scientific]) using a Beckmann Coulter SW41 Ti rotor at 38,000 rpm (239,000*g*) at 4°C for 2.5 h. For profiles of RNase-digested samples (Wu et al. 2020), lysates were treated with RNase A (Thermo Fisher Scientific) at 4 mg/L for 15 min at RT and quenched by adding 200 U of RiboLock RNase Inhibitor (Thermo Fisher Scientific). RNase-treated lysates were resolved through 10%–35% sucrose gradients using a Beckmann Coulter SW41 Ti rotor at 38,000 rpm (239,000*g*) for 4°C for 2 h. Gradients were fractionated using a Brandel Gradient fractionator and the absorbance at 254 nm was recorded.

### Western blot analysis

Samples were boiled in Bolt LDS Sample loading buffer (Thermo Fisher Scientific) containing Bolt/NuPAGE reduction buffer (Thermo Fisher Scientific) at 70°C for 10 min. Samples were resolved on 4%–12% Bolt Bis-Tris Plus protein gels (Thermo Fisher Scientific) using 1× Bolt MES or MOPS SDS running buffer (Thermo Fisher Scientific) containing 1× NuPAGE Antioxidant (Thermo Fisher Scientific), according to the manufacturer's instructions. Gels were transferred to nitrocellulose membranes using a Power Blot system (Thermo Fisher Scientific) using medium-range manufacturer parameters. Membranes were blocked with 5% nonfat dry milk (BioWorld) in PBST (10 mM Tris-HCl, pH 8 [Invitrogen], 1 mM EDTA [Invitrogen], 0.1% Triton X-100 [Sigma], 150 mM sodium chloride [Sigma]) for 1 h at room temperature (RT) with gentle rocking. Blots were washed in PBST three times and then incubated with the indicated primary antibodies overnight at 4°C with gentle rocking. Blots were washed with PBST three to four times over 45–60 min at RT with gentle rocking, then incubated with secondary antibodies diluted in 5% milk in PBST for 1 h with gentle rocking at RT. Membranes were washed again with PBST, three to four times over 45–60 min at RT, developed using SuperSignal West Pico Plus ECL substrate (Thermo Fisher Scientific) and SuperSignal West Femto Maximum Sensitivity substrate (Thermo Fisher Scientific) if needed and imaged on an iBright CL1000 (Thermo Fisher Scientific) system. Results shown are representative of at least two independent experiments.

### Phos-tag gels

For Phos-tag gel immunoblotting (Kinoshita et al. 2006), samples were resolved on homemade 12% Bis-Tris, pH 6.8, SDS–PAGE gels containing 50 µM Phos-tag (Wako, AAL-107), and 100 µM ZnCl_2_. Samples were boiled in Bolt LDS Sample loading buffer (Thermo Fisher Scientific) containing Bolt/NuPAGE reduction buffer (Thermo Fisher Scientific) at 70°C for 10 min. Samples were resolved using 1× Bolt MES SDS running buffer (Thermo Fisher Scientific) containing 1× NuPAGE Antioxidant (Thermo Fisher Scientific), according to the manufacturer's instructions. Gels were run using a constant 90 V until the bromophenol blue dye reached the bottom buffer. Before transfer to the nitrocellulose membrane, gels were soaked in 1 mM EDTA for 10 min with agitation to remove the Zn^+^ ions. Gels were transferred to nitrocellulose membranes using a Power Blot system (Thermo Fisher Scientific) using medium-range manufacturer parameters. Membranes were blocked with 5% nonfat dry milk (BioWorld) in PBST (10 mM Tris-HCl, pH 8 [Invitrogen], 1 mM EDTA [Invitrogen], 0.1% Triton X-100 [Sigma], 150 mM sodium chloride [Sigma]) for 1 h at RT with gentle rocking. Blots were washed in PBST three times and then incubated with indicated primary antibodies overnight at 4°C with gentle rocking. Blots were washed with PBST three to four times over 45–60 min at RT with gentle rocking, then incubated with secondary antibodies diluted in 5% milk in PBST for 1 h with gentle rocking at RT. Membranes were washed again with PBST three to four times over 45–60 min at RT, developed using SuperSignal West Pico Plus ECL substrate (Thermo Fisher Scientific) and SuperSignal West Femto Maximum Sensitivity substrate (Thermo Fisher Scientific) (if needed) and imaged on an iBright CL1000 (Thermo Fisher Scientific) system. Results shown are representative of at least two independent experiments. The percentages of the phosphorylation were estimated by comparing the intensities of the bands of phosphorylated and not phosphorylated forms of factors using ImageJ software (Schneider et al. 2012). The background in the gel images was estimated using a rectangular region with equal dimensions positioned immediately above the band corresponding to the phosphorylated form of the proteins. A similar rectangular region was also selected below the nonphosphorylated band.

### Antibodies

The following antibodies were used in this study. Antibodies for eIF2α (9722S), phospho-eIF2α (S51) (9721S), eEF2 (2332S), and phospho-eEF2 (T56) (2331S) were from Cell Signaling Technology. Antibodies for RPS19 (A304-002A) were purchased from Bethyl Laboratories Inc. Anti-mouse IgG-HRP (sc-525409), eIF2Bε (sc-55558), and GADD34 (sc-373815) were from Santa Cruz Biotechnology. Anti-rabbit ECL IgG-HRP (NA934V) was from Thermo Fisher Scientific.

## SUPPLEMENTAL MATERIAL

Supplemental material is available for this article.

## Figures and Tables

**Nikolay Aleksashin:**
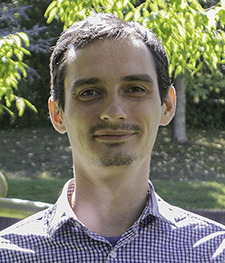


## MEET THE FIRST AUTHOR

***Meet the First Author(s)* is an editorial feature within *RNA*, in which the first author(s) of research-based papers in each issue have the opportunity to introduce themselves and their work to readers of *RNA* and the RNA research community. Nikolay Aleksashin is the first author of this paper, “A highly efficient human cell-free translation system.” He is a PhD postdoc in Jamie Cate's laboratory at the Innovative Genomics Institute, University of California, Berkeley. He is interested in the structural and functional characterization of human protein biosyntheses.**

**What are the major results described in your paper and how do they impact this branch of the field?**

The major results of this study involve the development of an optimized and highly efficient human cell-free protein synthesis (CFPS) system. This novel CFPS system utilizes extracts from human HEK293T cells, expressing GADD34 and K3L proteins, which effectively suppress the phosphorylation of the translation initiation factor eIF2α. The key innovation lies in the process of overexpressing GADD34 and K3L proteins in human cells before lysate preparation, streamlining the overall procedure. The study demonstrates that this improved CFPS system enhances the translation of mRNA molecules that rely on 5′ cap-dependent initiation as well as those utilizing internal ribosome entry site (IRES) mediated translation initiation. These results have significant implications for the fields of molecular and structural biology. The optimized CFPS system offers a powerful tool for researchers to explore human translation mechanisms under more physiologically relevant conditions outside of the cell. This can lead to a deeper understanding of the intricacies of protein synthesis, enabling scientists to investigate a wide range of scientific questions related to gene expression, protein function, and regulation. Additionally, the enhanced efficiency of this CFPS system holds promise for various applications in biotechnology, including the rapid production of therapeutic proteins and the study of disease mechanisms.

**What led you to study RNA or this aspect of RNA science?**

My journey into the world of RNA science was catalyzed by a combination of scientific fascination and the realization of RNA's pivotal role in the intricate machinery of life. Initially, I was drawn to this field by the captivating idea that RNA molecules, once regarded solely as intermediaries in the flow of genetic information, held an array of functions far beyond their initial description. The burgeoning research in noncoding RNAs, ribosomes, and the crucial regulatory roles of RNA in gene expression profoundly intrigued me. Furthermore, witnessing the groundbreaking discoveries and advancements in RNA science, particularly its relevance in various diseases and therapeutic applications, solidified my commitment to this area of study. The potential to uncover novel insights into cellular processes, the promise of developing innovative therapies, and the opportunity to contribute to the evolving landscape of molecular biology have all been driving forces in my pursuit of RNA science.

**What are some of the landmark moments that provoked your interest in science or your development as a scientist?**

Several landmark moments have significantly fueled my passion for science and shaped my journey as a scientist. One pivotal experience was during my early education when I conducted a simple experiment at a school science fair and witnessed the wonder of scientific discovery firsthand. This moment sparked my curiosity and instilled a deep interest in the scientific process. Another defining moment was when I had the opportunity to work as an intern in a research laboratory during my undergraduate studies. Collaborating with experienced scientists, I not only gained practical skills but also understood the profound impact that scientific research can have on society. Additionally, attending scientific conferences and hearing from esteemed researchers in my field provided valuable insights and inspiration. These collective experiences have been instrumental in nurturing my enthusiasm for science and my growth as a scientist, motivating me to pursue further studies and contribute to the scientific community.

**If you were able to give one piece of advice to your younger self, what would that be?**

If I had the opportunity to impart one piece of advice to my younger self, it would undoubtedly be to embrace kindness, unwavering self-belief, and the tenacity to persevere without surrender. In the journey of life, there will be challenges, moments of self-doubt, and obstacles that seem insurmountable. Ultimately, it's persistence and faith in one's potential that pave the path to personal growth and achievement. So, to my younger self, I would say, “Be kind, believe in yourself, and keep going—never give up.”

**Are there specific individuals or groups who have influenced your philosophy or approach to science?**

The formation of my scientific philosophy and approach to science has been profoundly shaped by my mentors, colleagues, and friends. These remarkable individuals have provided me with a wealth of knowledge, guidance, and inspiration, allowing me to develop a well-rounded perspective on the world of science. Their unwavering support and constructive critiques have been instrumental in my growth as a scientist. Through their diverse experiences, I have learned the value of collaboration, the importance of interdisciplinary thinking, and the significance of fostering a sense of camaraderie within the scientific community. My mentors have shared their wisdom and expertise, my colleagues have challenged and expanded my ideas, and my friends have infused the journey with shared enthusiasm and encouragement. Together, they have helped me navigate the intricate landscape of scientific exploration and have been indispensable in shaping my scientific journey.
